# Effects of Behavioral Economics–Based Messaging on Appointment Scheduling Through Patient Portals and Appointment Completion: Observational Study

**DOI:** 10.2196/34090

**Published:** 2022-03-30

**Authors:** Su-Ying Liang, Cheryl D Stults, Veena G Jones, Qiwen Huang, Jeremy Sutton, Guy Tennyson, Albert S Chan

**Affiliations:** 1 Sutter Health Center for Health Systems Research Palo Alto, CA United States; 2 Clinical Leadership Team Sutter Health Sacramento, CA United States; 3 Center for Biomedical Informatics Research Stanford University Stanford, CA United States

**Keywords:** access to care, behavioral economics, online, web-based appointment scheduling, health service, behavior, health care

## Abstract

**Background:**

Behavioral economics–based techniques have been an increasingly utilized method in health care to influence behavior change by modifying language in patient communication (through choice architecture and the framing of words). Patient portals are a key tool for facilitating patient engagement in their health, and interventions deployed via patient portals have been effective in improving utilization of preventive health services.

**Objective:**

We examined the impacts of behavioral economics–based nudge health maintenance reminders on appointment scheduling through a patient portal and appointment completion for 2 preventive services: Medicare wellness visits and Pap smear.

**Methods:**

We conducted a retrospective observational study using electronic health record data from an integrated health care system in Northern California. Nudge health maintenance reminders with behavioral economics–based language were implemented for all sites in November 2017 for Medicare wellness visits and for selected sites in February 2018 for Pap smears. We analyzed 125,369 health maintenance reminders for Medicare wellness visits, and 585,358 health maintenance reminders for Pap smear sent between January 2017 and February 2020. The primary outcomes were rate of appointments scheduled through the patient portal and appointment completion rate. We compared the outcomes between those who received the new, behavioral economics–based health maintenance reminders (the nudge group) and those who received the original, standard health maintenance reminders (the control group). We used segmented regression with interrupted time series to assess the immediate and gradual effect of the nudge for Medicare wellness visits, and we used logistic regression to assess the association of nudge health maintenance reminders, adjusting for the propensity to receive a nudge health maintenance reminder, for Pap smear.

**Results:**

The rates of appointments scheduled through the patient portal were higher for nudge health maintenance reminder recipients than those for control health maintenance reminder recipients (Medicare wellness visits—nudge: 12,537/96,839, 13.0%; control: 2,769/28,530, 9.7%, *P*<.001; Pap smear—nudge: 8,239/287,149, 2.9%; control: 1,868/120,047, 1.6%; *P*<.001). Rates of appointment completion were higher for nudge health maintenance reminders for Pap smear (nudge: 67,399/287,149, 23.5% control: 20,393/120,047, 17.0%; *P*<.001) but were comparable for Medicare wellness visits (nudge: 49,835/96,839, 51.5% control: 14,781/28,530, 51.8%; *P*=.30). There was a marginally gradual effect of nudge on number of appointments scheduled through the patient portal for the overall Medicare wellness visits sample (at a monthly rate of 0.26%, *P*=.09), and a significant gradual effect among scheduled appointments (at a monthly rate of 0.46%, *P*=.04). For Pap smear, nudge health maintenance reminders were positively associated with number of appointments scheduled through the patient portal (overall sample: propensity adjusted odds ratio [OR] 1.62; 95% CI 1.50-1.74; among scheduled appointments: propensity adjusted OR 1.61, 95% CI 1.47-1.76) and with appointment completion (propensity adjusted OR 1.07; 1.04-1.10).

**Conclusions:**

Nudges, a behavioral economics–based approach to providing health maintenance reminders, increased the number of appointments scheduled through the patient portal for Medicare wellness visits and Pap smear. Our study demonstrates that a simple approach—framing and modifying language in an electronic message—can have a significant and long-term impact on patient engagement and access to care.

## Introduction

Health care has become more directly accessible to the patient, in part due to the Health Information Technology for Economic and Clinical Health Act of 2009 [1], and also, as a result of increasing consumer demands [2].When first introduced, patient portals provided patients with limited access to their medical records and adoption was low [3]. In part to meet meaningful use requirements of the Health Information Technology for Economic and Clinical Health Act, web-based electronic health record portal capabilities have expanded to appointment scheduling, displaying lab results or viewing encounter notes written by their physician [4], allowing bills payments, and facilitating communication with care teams, costs estimates for ambulatory services [5], and access to family records. Patient access rates to web-based patient portals have increased to 90% in some organizations [6]. Yet, access alone can only go so far in engaging patients with patient portals. Message construction and delivery also influence patient utilization of patient portals [7].

In the field of behavioral economics, researchers recognize that humans do not always act logically [8] in making choices and provide tools to help influence desired behaviors. One strategy includes choice architecture interventions, in which the presentation of options are altered to improve decision-making without restricting choice [9]. Subtle design changes, such as reordering choices, limiting options, or modifying a default can significantly influence behavior. When applying these tools and others, choice architects operate with a key tenet in mind: reduce friction in decision-making to make the desired path the one of least resistance [8,10]. In addition to the choice environment, behavioral economists also know that the framing of words can nudge individuals in a given direction of action [9]. For instance, messages that use common language [11], harness aversion to loss and regret [11,12], or embed social elements and devices [11-13] into their core are likely to be very effective at driving behavior change [11-13].

Over the past decade, these tenets of behavioral economics have been increasingly utilized in health care for both patients (cancer screenings [14], hospital appointment no-show rates [7], medication adherence for behavioral health [15], HIV testing rates [16], obesity and binge eating [17]) and clinicians (prescribing behavior [18,19], overtreatment of diabetes [20]), with 83 publications in the top 3 highest impact general medicine journals (Journal of the American Medical Association, The Lancet, The New England Journal of Medicine) from 1998 to 2018 [21]. A systematic review [22] found patient portal interventions to be effective in improving a few psychological outcomes, medication adherence, and preventive service use. Yet, to the best of our knowledge, behavioral economics has not been applied to health maintenance reminders sent through web-based patient portals to improve patient engagement and increase preventive service use for annual Medicare wellness visits and Pap smear. General use of the Medicare wellness visits has increased over time [23], with 7% [24] to 8.1% [25] of Medicare beneficiaries receiving an annual wellness visit in 2011 increasing to 16% in 2014 [24] and 23% in 2016 [25]. Although rates for Pap smear are much higher, with 83.7% of women age 21 to 65 years reporting having one within the past 3 years in 2018 [26], there is still room for improvement. Our objective was to determine if small changes in the wording of health maintenance reminders could alter patient completion of these preventive health services.

## Methods

### Setting

Sutter Health is a large not-for-profit health care system serving more than 3 million people annually across 100 rural and urban communities in Northern California. Sutter Health was the first health care system in the United States to implement Epic System’s MyChart patient portal (My Health Online) in 2001 [27,28]. As of 2020, there have been over 3 million patients enrolled in My Health Online, which can be accessed via the website or through the mobile app, to communicate with their care team, refill prescriptions, view lab results, pay bills, and schedule appointments (including video visits).

### Pilot Testing: Behavioral Economics–Based Email Messaging

In September 2017, we conducted pilot testing to assess the application and effectiveness of behavioral economics–driven language in encouraging patients to schedule appointments using the patient portal with the 2 largest of the 5 Sutter Health medical foundations (Palo Alto Medical Foundation and Sutter Medical Foundation) in the Sacramento Valley. There were 775,000 Palo Alto Medical Foundation patients enrolled in My Health Online with 222,000 unique log-ins in September 2017 and 41,000 appointments scheduled through the patient portal. There were 436,000 Sutter Medical Foundation patients enrolled in My Health Online, with 103,000 unique log-ins in September 2017 and 13,000 appointments scheduled through the patient portal. We selected 2 sites to demonstrate that nudge health maintenance reminder could be useful for more than one patient population. This also allowed us to understand potential confounders and variables before scaling across the organization.

**Figure 1:**
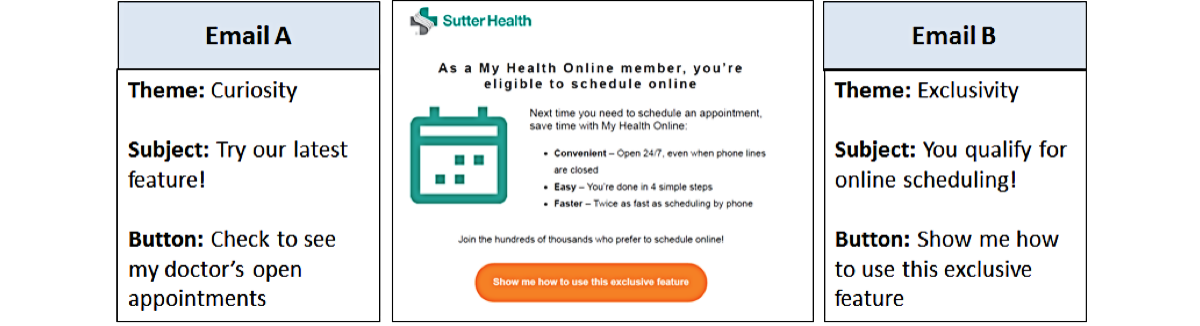
Themes of pilot behavioral economics–based email messages.

The pilot consisted of testing of 2 message options, followed by the roll-out of the message for which the most patients scheduled appointments through the patient portal. We tested 2 themes—curiosity (email A) and exclusivity (email B)—in patient messaging (Figure 1). We selected these themes based on guidance from behavioral economics experts at VAL Health based upon their previous experiences [29-31]. Among 550,000 My Health Online members from Palo Alto Medical Foundation and Sutter Medical Foundation who had never scheduled appointments through the patient portal, 3800 patients were randomized to receive the promotional emails. More patients, of both Sutter Medical Foundation and Palo Alto Medical Foundation, scheduled appointments through the patient portal after receiving email B, thus email B was selected for use in the next phase.

Email B was sent to the remaining Palo Alto Medical Foundation and Sutter Medical Foundation members who had never scheduled appointments through the portal (n=550,000) on November 10, 2017. We included a control group of 10% of Palo Alto Medical Foundation and Sutter Medical Foundation My Health Online users to see if there was any difference in rates of appointments scheduled through the patient portal compared to those for individuals who received nudge emails.

### Application to Health Maintenance Reminders

**Figure 2:**
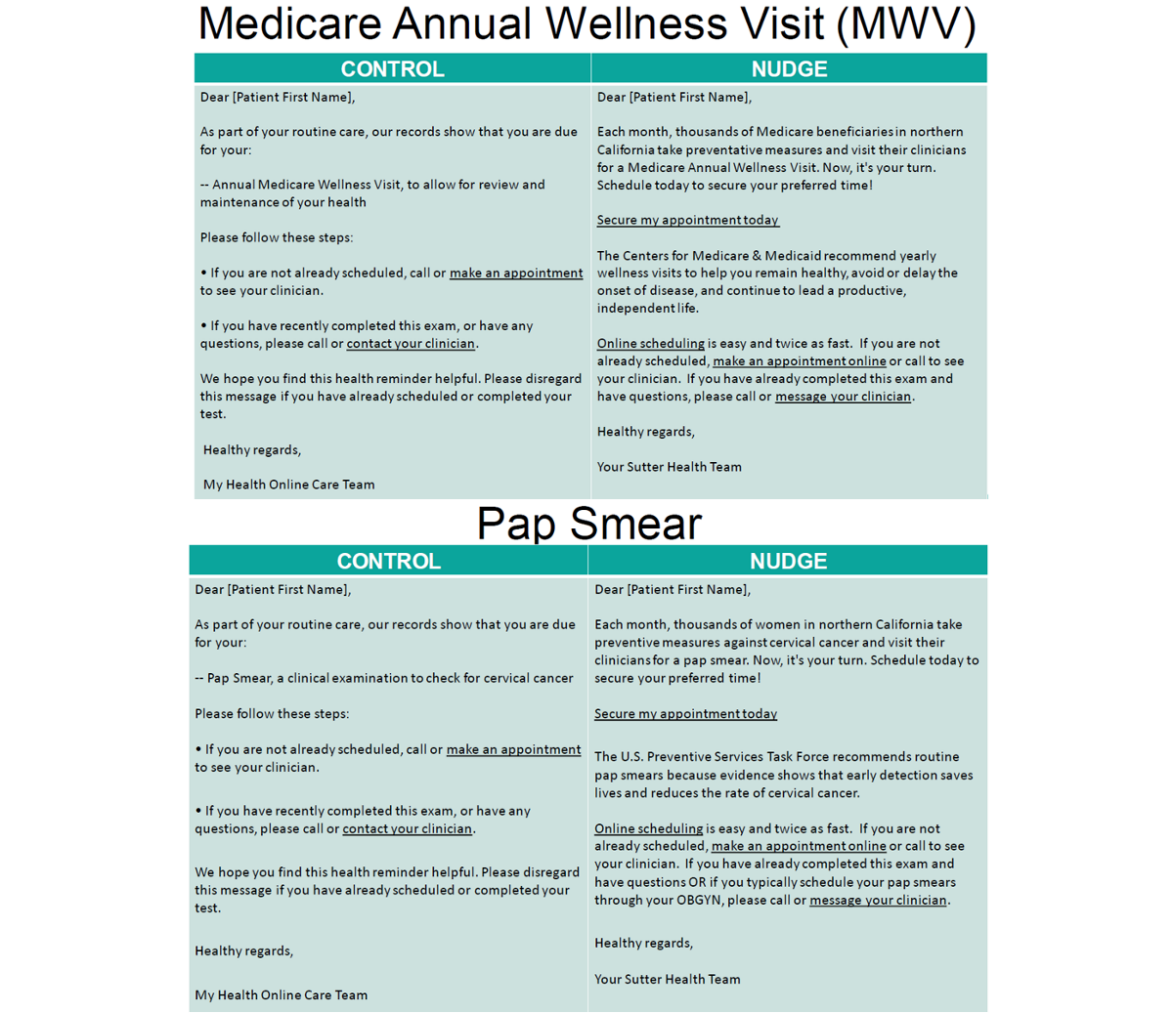
Standard (control) and behavioral economics–based (nudge) health maintenance reminders.

**Figure 3:**
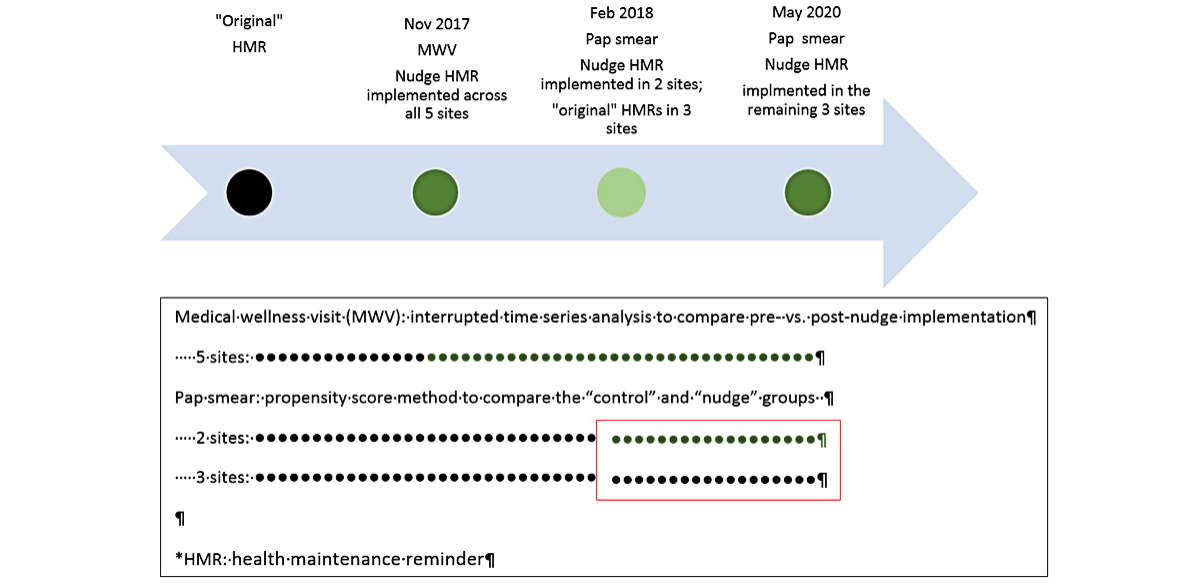
Study design. HMR: health maintenance reminder; MWV: Medicare wellness visit.

The same behavioral economics concept was subsequently applied to health maintenance reminder messages in the patient portal. Nudge message wording was redesigned to use the exclusivity theme, with embedded functionality to click to schedule appointments through the patient portal for Medicare wellness visits and Pap smear (Figure 2). We selected Medicare wellness visits because it was already part of an active initiative at Sutter Health to increase utilization and we hoped that this type of message would better connect with patients who were eligible for Medicare wellness visits. We selected Pap smear so that we could examine how scheduling functions may impact screening completion and disease prevention. Nudge health maintenance reminders for Medicare wellness visits were implemented for all 5 Sutter medical foundations on November 15, 2017 (Figure 3). Nudge health maintenance reminders for Pap smear were launched on February 18, 2018; patients at Palo Alto Medical Foundation and Sutter Medical Foundation received nudge health maintenance reminders, while patients at the other 3 Sutter medical foundations continued to receive standard health maintenance reminders (control). Health maintenance reminders for Medicare wellness visits and Pap smear were discontinued during the COVID-19 pandemic; therefore, we analyzed utilization between January 2017 and February 2020.

### Statistical Analysis

#### Study Sample

For this retrospective, observational study with electronic health record data, we identified 125,369 Medicare wellness visit health maintenance reminders sent to 43,889 unique patients who were 65 years and older between January 1, 2017 and February 28, 2020, and we identified 585,358 health maintenance reminders for Pap smear sent to 288,152 unique patients who were 21 years and older during the same study period.

#### Measures and Statistical Approach

The primary outcomes were rates of appointments scheduled through the patient portal and completed. The predictor of interest in all analyses was the receipt of nudge health maintenance reminder. The 2-tailed *t* test was used to examine differences in the unadjusted proportions of appointments scheduled through the patient portal and appointment completion between the nudge and control groups. We also conducted a subgroup analysis focusing on patterns of proportion of appointments scheduled through the patient portal among scheduled appointments.

For Medicare wellness visits, we used segmented regression with interrupted time series analysis to assess the immediate and gradual impact of the nudge intervention on study outcomes. The advantage of this analytic approach is its ability to distinguish the intervention effect from the secular trend (ie, a trend change that would have happened even in the absence of the intervention). The unit of analysis was the month. Study outcomes were measured in a given month: percentage of appointments scheduled through the patient portal (*number of health maintenance reminders leading to appointment scheduled online* / *total number of health maintenance reminders*) and percentage of appointment completion (*number of health maintenance reminders with appointment completed* / *total number of health maintenance reminders*).

For Pap smear, after initial launch of nudge health maintenance reminders, there remained a mix of nudge and control health maintenance reminders between February 2018 and February 2020. We used logistic regression models to assess the association of nudge health maintenance reminders with study outcomes, adjusting for the propensity to receive a nudge health maintenance reminder and accounting for clustering within the patients. The unit of analysis was the health maintenance reminder. The outcomes were use of the patient portal to schedule an appointment (yes or no) and completion of the appointment (yes or no). The propensity score method was chosen to control for potential selection bias and confounding by factors associated with the intervention and the study outcomes [32,33]. We estimated the propensity to receive a nudge health maintenance reminder as a function of patient demographic characteristics (age and race/ethnicity: White, Black, Hispanic, Asian, other), Charlson comorbidity score at baseline, health care utilization (number of health care encounters including in-person visit, video visits, My Health Online messages, and telephone calls) in the 12 months prior to baseline, insurance type (preferred provider organization or fee-for-service, health maintenance organization, Medicare, Medicaid, other), and their primary care physician’s service location. Baseline was defined as the date of the first health maintenance reminder that the patient received during the analysis timeframe. Linear and categorical specifications of the propensity score were evaluated to ensure the robustness of the results. Analyses were conducted in R (version 4.0.4; The R Project) and Stata (version 16.1; StataCorp LLC).

### Ethics

The study was reviewed by the Sutter Health Institutional Review Board (SHIRB) and approved as a quality improvement study (IORG0004135).

## Results

### Sample Characteristics

Of 125,369 health maintenance reminders sent for Medicare wellness visits, 77% (96,839) were nudge health maintenance reminders (ie, using behavioral economics–based language). Of 125,369 Medicare wellness visits health maintenance reminder portal messages sent, 60.2% (75,407) led to an appointment being scheduled, with 12.2% (15,342) scheduled through the patient portal, and 51.5% (64,616) appointments completed. Of 585,358 health maintenance reminders sent for Pap smear, 49.1% (287,149) were nudge health maintenance reminders. Of 585,358 Pap smear health maintenance reminder portal messages sent, 21.9% (128,329) led to appointment being scheduled, 2.3% (13,259) scheduled through the patient portal, and 21.9% (128,255) appointments completed (Table 1).

Of 43,889 patients (age: mean 75 years, SD 7.2) included in the Medicare wellness visit analysis, 60.7% (26,662/43,889) were women. Approximately two-thirds (33,837/43,889, 77.1%) were White, and 41% (18,175/43,889) had no comorbid conditions. The mean number of encounters in the 12 months prior to the baseline date was 22 (SD 20.6). Of 288,152 women (age: mean 41 years, SD 12.8) included in the Pap smear analysis, there were diverse racial/ethnic groups (White: 141,426/288,152, 49.1%; Black: 8919/288,152, 3.1%; Hispanic: 37,398/288,152, 13.0%; Asian: 59,225/288,152, 20.6%; other: 41,184/288,152, 14.3%); 89% (255,796/288,152) had no comorbid conditions. The mean number of encounters in the 12 months prior to baseline was 7 (SD 12.0); 45.9% (132,151/288,152) had coverage through preferred provider organization or fee-for-service plans, and 14.4% (41,618/288,152) had coverage through health maintenance organization plans (Table 2).

**Table 1:** Health maintenance reminders sent and outcomes.

Health maintenance reminder						Medicare wellness visits (n=125,369), n (%)			Pap smear (n=585,358), n (%)
**Type**									
	Nudge					96,839 (77.2)			287,149 (49.1)
	Control					28,530 (22.8)			298,209 (50.9)
**Appointment scheduled**									
	No					49,962 (39.8)			457,029 (78.1)
	Yes					75,407 (60.2)			128,329 (21.9)
		**Scheduled through patient portal**							
			Yes	15,342 (12.2)			13,259 (2.3)		
			No	110,027 (87.8)			572,099 (97.7)		
		**Appointment completed**							
			Yes	64,616 (51.5)			128,255 (21.9)		
			No	60,753 (48.5)			457,103 (78.1)		

**Table 2:** Sample characteristics.

Characteristic				Medicare wellness visits (n=43,889), n (%)		Pap smear (n=288,152), n (%)
Age (years), mean (SD)				75.3 (7.2)		40.9 (12.8)
**Gender, n (%)**						
	Male			17,267 (39.3)		0 (0)
	Female (%)			26,622 (60.7)		288,152 (100)
**Race/ethnicity, n (%)**						
	White			33,837 (77.1)		141,426 (49.1)
	Black			1121 (2.6)		8919 (3.1)
	Hispanic			2947 (6.7)		37,398 (13.0)
	Asian			3476 (7.9)		59,225 (20.6)
	American Indian or Alaska Native/Pacific Islander or Native Hawaiian			198 (0.4)		1639 (0.6)
	Multirace			347 (0.8)		5819 (2.0)
	Unknown			1963 (4.5)		33,726 (11.7)
**Comorbidity score at baseline, n (%)**						
	0			18,175 (41.4)		255,796 (88.8)
	1			8462 (19.3)		22,167 (7.7)
	2			7027 (16.0)		6882 (2.4)
	3+			10,225 (23.3)		3307 (1.1)
**Health care utilization at baseline, n (%)**						
	Encounters^a^			22.3 (20.6)		6.6 (12.0)
	**Insurance type, n (%)**					
		Health maintenance organization	373 (0.9)		41,618 (14.4)	
		Medicaid or Medi-Cal	36 (0.1)		9713 (3.4)	
		Medicare fee-for-service	28,291 (64.4)		7626 (2.7)	
		Medicare health maintenance organization	14,168 (32.3)		1045 (0.4)	
		Preferred provider organization or fee-for-service	750 (1.7)		132,151 (45.9)	
		Other/Unknown	271 (0.6)		95,999 (33.2)	
	**Region (primary care physician’s location at baseline), n (%)**					
		Region A	12,864 (29.3)		143,518 (49.8)	
		Region B	4133 (9.4)		19,969 (6.9)	
		Region C	24,402 (55.6)		62,988 (21.9)	
		Region D	1681 (3.8)		18,587 (6.5)	
		Region E	763 (1.7)		16,966 (5.8)	
		Other or no primary care physician	46 (0.1)		26,135 (9.1)	

^a^Encounters included in-person visits, video visits, My Health Online message, and telephone.

### Appointment Scheduling and Completion

**Figure 4:**
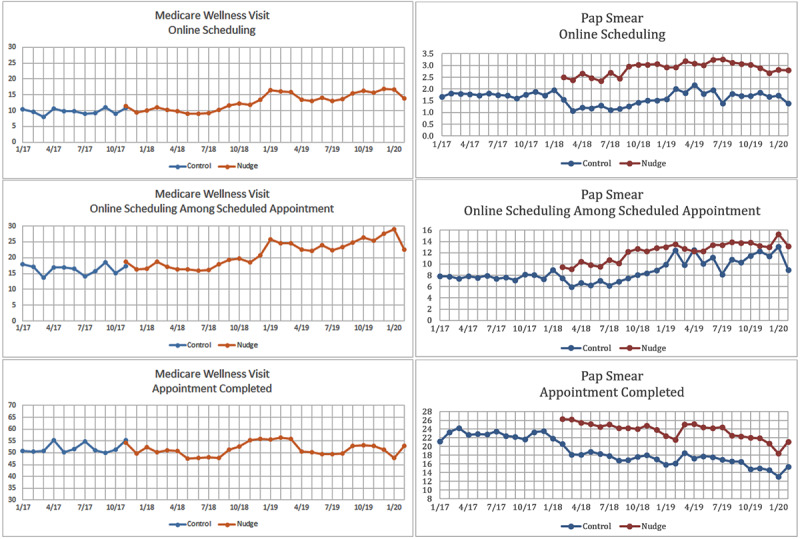
Trends of web-based scheduling and appointment completion from January 2017 to February 2020.

For Medicare wellness visits, there was an increasing trend in proportion of appointments scheduled through the patient portal (Figure 4), and for Pap smear, appointments scheduled through the patient portal and appointment completion in the nudge group were consistently higher than those for the control group throughout the study period.

When comparing the unadjusted rates, we observed that a higher percentage of patients scheduled Medicare wellness visits through the patient portal after nudge implementation (nudge) than before implementation (control) for the overall sample (nudge: 12,573/96,839, 13.0%; control: 2769/28,530, 9.7%; *P*<.001) as well as for the subset with appointment scheduled (nudge: 12,573/58,371, 21.5%, control: 2769/17,036 16.3%; *P*<.001) (Table 3). A similar pattern for appointment scheduling through the patient portal was found for Pap smear between February 2018 and February 2020 for those who received health maintenance reminders (nudge: 8239/287,149, 2.9%, control: 1868/120,047, 1.6%; *P*<.001) and for the subset with appointment scheduled (nudge: 12.2%, control: 9.2%, *P*<.001), and the rate of appointment completion for Pap smear was higher (*P*<.001) in the nudge group (67,399/287,149, 23.5%) than that in the control group (20,393/120,047, 17.0%), while rates of appointment completion for Medicare wellness visits were comparable (nudge: 49,835/96,839, 51.5%; control: 14,781/28,530, 51.8%; *P*=.30).

**Table 3:** Rates of appointment scheduling through the patient portal and appointment completion between nudge and control groups.

					Medicare wellness visits						Pap smear				
				Control (January 2017-November 2017)		Nudge (December 2017-February 2020)		*P* value		Control (February 2018-February 2020)		Nudge (February 2018-February 2020)		*P* value	
**Health maintenance reminders, n**					28,530		96,839		—^a^		120,047		287,149		—
	**Appointments scheduled, n**			17,036		58,371		—		20,402		67,445		—	
		**Appointment scheduled through the patient portal, n**		2769		12,573		—		1868		8239		—	
			Unadjusted^b^, %	9.7		13.0		<.001		1.6		2.9		<.001	
			Adjusted^c^, %	16.3		21.5		<.001		9.2		12.2		<.001	
	Appointments completed, n (%)			14,781 (51.8)		49,835 (51.8)		.30		20,393 (17.0)		67,399 (23.5)		<.001	

^a^The comparison was not made.

^b^The percentage was calculated using the number of health maintenance reminders.

^c^The percentage was calculated using the number of appointments scheduled.

### Nudge Effect

Findings from segmented regression for Medicare wellness visits (Table 4) suggested that there was a marginal gradual effect of nudge messaging on Medicare wellness visits scheduled through the patient portal (monthly rate 0.26%; *P*=.09). There was a statistically significant increase, at a rate of 0.46% per month (*P*=.04), in appointments scheduled through the patient portal. There was no immediate effect for either patterns in appointments scheduled though the patient portal or for patterns in appointments completed for Medicare wellness visits.

The odds of scheduling a Pap smear for patients who received nudge health maintenance reminders were 1.62 times those for patients who received control health maintenance reminders (propensity adjusted odds ratio [OR] 1.62, 95% CI 1.50-1.74). A similar association was observed among the patients with scheduled appointments (propensity adjusted OR 1.61, 95% CI 1.47-1.76). Nudge health maintenance reminders were associated with a 7% increase in the odds of appointment completion for Pap smear (propensity adjusted OR 1.07, 95% CI 1.04-1.10).

We evaluated linear and categorical specifications (quintiles and deciles) of the propensity score. The categorical specification in deciles was selected, although sensitivity analyses using different specifications of the propensity score did not change our conclusions. Nudge health maintenance reminders were associated with a higher rate of scheduling through the patient portal for all health maintenance reminders and for patients who received health maintenance reminders and who scheduled appointments. For all health maintenance reminders, ORs ranged between 1.39-1.63 (quintile specification: adjusted OR 1.63, 95% CI 1.51-1.75; linear specification: adjusted OR 1.39, 95% CI 1.32-1.47). For patients with scheduled appointments, ORs ranged between 1.47-1.56 (quintile specification: adjusted OR 1.56, 95% CI 1.44-1.70; linear specification: adjusted OR 1.47, 95% CI 1.36-1.58). Nudge health maintenance reminders were associated with 10% to 17% increases in the odds of appointment completion for Pap smear (quintile specification: adjusted OR 1.17, 95% CI 1.14-1.20; linear specification: adjusted OR 1.10, 95% CI 1.07-1.12).

**Table 4:** Regression analysis results.

	Medicare wellness visits, coefficient (SE)					Pap smear^a^, odds ratio (95% CI)	
	Preintervention level (Baseline)	Preintervention slope (secular trend, per month)	Change in intercept (immediate effect)	Change in slope (gradual effect, per month)	Unadjusted		Propensity adjusted
Scheduled through the patient portal	9.421 (0.941)	0.001 (0.151)	−0.700 (0.971)	0.264 (0.155)	1.87 (1.78 to 1.97)		1.62 (1.50 to 1.74)
Scheduled through the patient portal among all scheduled	16.343 (1.305)	−0.043 (0.210)	−1.071 (1.345)	0.462 (0.215)	1.38 (1.31 to 1.46)		1.61 (1.47 to 1.76)
Appointment completed	50.687 (1.728)	−0.007 (0.279)	−0.201 (1.782)	0.052 (0.285)	1.50 (1.47 to 1.53)		1.07 (1.04 to 1.10)

^a^The reference group in the model is patients who received a standard (control) health maintenance reminder. The propensity to receive a nudge health maintenance reminder was estimated as a function of patient age at baseline, race/ethnicity, Charlson comorbidity score at baseline, number of encounters at baseline, insurance type, and service location and was categorized in deciles in the adjusted model.

## Discussion

### Principal Findings

To the best of our knowledge, this is the first attempt to employ behavioral economics with electronic medical record portal–based health maintenance reminders to improve patient engagement and the utilization of preventive health services. Our findings suggest that our intervention influenced patient behavior. Simple modifications to verbiage and the framing of messages and adding embedded scheduling functionality had an impact on patients’ actions. Although we did not find an immediate effect of nudge on scheduling appointments through the patient portal, we observed a sustained effect over time especially among those who scheduled an appointment. We believe this is because once patients learned how to schedule appointments through the patient portal, they used the portal to schedule appointments from that time onward. Similarly, we found that nudge health maintenance reminders for Pap smear were associated with a 65% to 68% increase in the odds of scheduling appointments through the patient portal, compared to control health maintenance reminders during the same study timeframe.

Appointments have typically been scheduled over the phone or in person, which can be resource intensive. Patient portals with web-based scheduling improves patient convenience, flexibility, and communications with providers, while reducing administrative burdens [34,35]. Furthermore, the COVID-19 pandemic has increased preference for and use of telehealth and features such as web-based scheduling. Behavioral economics–based health maintenance reminders are low-cost, effective, and operationally feasible. Once designed and pilot tested, they can be centrally deployed to all eligible patients with relatively low administrative burden and costs.

We acknowledge the very low rate for scheduling appointments through the patient portal for Pap smear (13,259/585,358, 2.3%) and posit that this may be due to a couple of factors. First, at Sutter Health, the majority of obstetric and gynecological practices do not allow appointments to be scheduled through the patient portal. Patients could schedule appointments with their primary care provider through the portal but could only type “Pap smear” in the free text box if they would like to receive one during that visit. Since there is no Pap smear visit scheduling type in the electronic medical record system, it added some complexity in retrieving these data—we identified the Pap smear procedure in subsequent visits. Nevertheless, even a small percentage means that more patients than those who would have with a standard message were able to receive this preventative screening.

Proactively engaging patients to schedule their appointments is the important first step to care management. Follow-through appointment completion is next. Our findings are mixed when examining the effect of nudge on appointment completion. We did not observe a significant effect on appointment completion for Medicare wellness visits (*P*=.30). The nudge effect for Pap smear was moderate, with a 7% increase, compared to control health maintenance reminders. The difference in nudge effects between Medicare wellness visits and Pap smear may be attributable to differences in patient populations to some degree. Exploratory stratified analyses suggested that patients who were younger (vs those 65 years and older) and Asian (vs other race or ethnicity groups) had the highest Pap smear appointment completion rate and most improvement from receiving nudge messages. Compared to the Medicare wellness visit sample, the Pap smear sample represented a younger patient population, with relatively more Asian individuals. We also explored if the number of health maintenance reminders sent plays a role in appointment completion. Among those who received multiple health maintenance reminders, subsequent health maintenance reminders were associated with higher rates of appointment completion than those for the first health maintenance reminders. These patterns were similar for both Medicare wellness visits and Pap smear. Scheduling an appointment or procedure does not automatically lead to completion, and thus, completion is a more complex process than scheduling and likely to be influenced by additional factors, which requires further investigation.

### Limitations

Our study has several limitations. First, our analyses are based on data from a large health care system in Northern California. Our nudge intervention was designed for patients with access to internet and email which limits the generalizability of specific estimates of the nudge effect. Second, the propensity score covariate–adjusted method was selected because it mitigates confounding in observational studies such as ours. We chose this method over other widely used propensity score–matching methods. Due to the case-control ratio in our data (approximately 2.4:1 between February 2018 and February 2020), the propensity score–matching approach would have resulted in a loss of statistical power. The rollout of nudge health maintenance reminders was designed with operational goals to encourage patient uptake of web-based tools and to facilitate scheduling, which is expected to lead to better care management and health outcomes. As such, more cases than controls were enrolled. Different specifications of propensity score modeling may affect the results of our propensity score covariate–adjusted approach. We conducted sensitivity analyses using linear and nonlinear specifications of propensity score and our findings were consistent. Third, we focused on the overall patterns. Future research is needed to understand potential variability by specific subgroups (race/ethnicity, socioeconomic status) to inform targeted, culturally appropriate designs to maximize the benefit of nudges. The time of the year may have played a role in appointment scheduling and completion. Our study period limited the ability to examine the seasonal effect. We also recognize that the generalizability of these findings might be limited, with respect to application to other patient portals, as many have different functionalities and user experiences than those in the portal used in this study.

### Conclusions

Nudges, a behavioral economics–based health maintenance reminder, improve web-based scheduling and subsequent appointment completion for Medicare wellness visits and Pap smear, with important long-term impacts. Given these results, Sutter Health implemented messages with behavioral economics–based language for all other health maintenance reminders on May 28, 2020. Future studies should explore why nudge worked for some patients and not others.

## Abbreviations

OR: odds ratio
